# Prediction of Bioluminescent Proteins Using Auto Covariance Transformation of Evolutional Profiles

**DOI:** 10.3390/ijms13033650

**Published:** 2012-03-19

**Authors:** Xiaowei Zhao, Jiakui Li, Yanxin Huang, Zhiqiang Ma, Minghao Yin

**Affiliations:** 1 School of Computer Science and Information Technology, Northeast Normal University, Changchun 130117, China; E-Mails: zhaoxw303@nenu.edu.cn (X.Z.); lijk136@126.com (J.L.); 2 School of Life Sciences, Northeast Normal University, Changchun 130024, China; 3 National Engineering Laboratory for Druggable Gene and Protein Screening, Northeast Normal University, Changchun 130024, China

**Keywords:** bioluminescent proteins, position specific scoring matrix, support vector machine, evolutionary information

## Abstract

Bioluminescent proteins are important for various cellular processes, such as gene expression analysis, drug discovery, bioluminescent imaging, toxicity determination, and DNA sequencing studies. Hence, the correct identification of bioluminescent proteins is of great importance both for helping genome annotation and providing a supplementary role to experimental research to obtain insight into bioluminescent proteins’ functions. However, few computational methods are available for identifying bioluminescent proteins. Therefore, in this paper we develop a new method to predict bioluminescent proteins using a model based on position specific scoring matrix and auto covariance. Tested by 10-fold cross-validation and independent test, the accuracy of the proposed model reaches 85.17% for the training dataset and 90.71% for the testing dataset respectively. These results indicate that our predictor is a useful tool to predict bioluminescent proteins. This is the first study in which evolutionary information and local sequence environment information have been successfully integrated for predicting bioluminescent proteins. A web server (BLPre) that implements the proposed predictor is freely available.

## 1. Introduction

Bioluminescence is a process in which light is produced in an organism by means of a chemical reaction [1,2]. Bioluminescence has been found in various organisms like squid, bacteria, fungi, ctenophore, algae and fish, *etc.* [3,4]. All bioluminescent reactions occur in the presence of oxygen. At least two chemicals are required in the bioluminescence process. The one which produces the light is genetically called a luciferin and the one that drives to catalyze the reaction is called a luciferase [5]. In the basic reaction, the luciferase catalyzes the oxidation of luciferin, resulting in light and an inactive oxyluciferin. In order to produce more luciferin, energy must be provided to the reaction system. Sometimes the luciferin and luciferase (as well as co-factor such as oxygen) are bound together in a single unit called a photoprotein. When a particular type of ion is added to the system, this molecule can be triggered to produce light.

Bioluminescence serves various functions, such as attraction of mates, attraction of prey, camouflage, finding food, signaling other members of their species and illumination of prey [3–5]. The application of bioluminescence can greatly promote the progress in the field of medical and commercial areas. Thus, identification of bioluminescent proteins could help to discover many still unknown functions and design new commercial and medical applications.

Until now, both experimental and computational methods [6,7] have been developed to investigate the bioluminescent proteins. But *in vitro* and *in vivo* methods are often time-consuming, expensive and have very limited scopes due to some restrictions for many enzymatic reactions. On the other hand, *in silico* prediction of bioluminescent proteins from computational approaches may provide fast and automatic annotations for candidate bioluminescent proteins. However, there are few studies using computational approaches to discriminate bioluminescent proteins and non-bioluminescent proteins. Kandaswamy *et al.* [8] have tried to solve this problem using support vector machine (SVM). To the best of our knowledge, that is the first and the only paper utilizing machine learning technique to deal with the prediction of bioluminescent proteins. With the model BLProt, they obtained 80% accuracy from training dataset and 80.06% accuracy from test dataset. A list of 544 physicochemical properties [9] was used to encode each protein sequence. The problem is worthy of further investigation because the prediction performance is not always satisfactory and there were no online web servers up until now.

In this study, we develop a new computational method to predict bioluminescent proteins. First, sequential evolution information in the form of position specific scoring matrix (PSSM) generated from the inquired sequences is obtained by PSI-BLAST. Second, the PSSM is transformed into a fixed-length feature vector by auto covariance (*AC*) transformation. This encoding strategy (PSSM-AC) has been successfully utilized to predict protein structural classes [10] and discriminate membrane proteins [11]. Finally, these resulting vectors are input to an SVM classifier to perform the prediction. Tested by 10-fold cross-validation and independent test, the accuracy of the proposed predictor reaches 85.17% for the training dataset and 90.71% for the testing dataset respectively, which are significantly higher than those by the existing predictors. We reckon that this efficient performance enhancement is largely due to the good discrimination capabilities of the feature extraction strategy PSSM-AC and the learning capabilities of SVM. The proposed predictor is freely accessible to the public at the web server BLPre [12].

## 2. Materials and Methods

### 2.1. Datasets

To evaluate the prediction model proposed in this study and compare it with state-of-the-art methods, two publicly available datasets are used here [8]. And anyone can freely download it at [13]. The training dataset contains 300 bioluminescent proteins and 300 non-bioluminescent proteins, and the test dataset contains 139 bioluminescent proteins and 18202 non-bioluminescent proteins.

To avoid homology bias and remove the redundant sequences from the benchmark dataset, a cutoff threshold of 25% is imposed by [14,15] to remove those proteins from the benchmark dataset that have ≥ 25% sequence similarity. However, we do not use such a stringent criterion in this study because the number of available protein sequences does not allow us to do so (40% in this paper). In addition, the protein sequences containing less than 50 amino acids are also screened out.

### 2.2. Position Specific Scoring Matrix

Evolutionary information, one of the most important types of information in assessing functionality in biological analysis, has been widely used in many studies [16–21]. To extract the evolutionary information, the profile of each protein sequence is generated by running Position Specific Iterated BLAST (PSI-BLAST) program [22–24]. Then this information can be represented as a two dimensional matrix which is known as the PSSM of the protein. PSSM has been widely used to predict protein fold pattern [25], protein quaternary structural attribute [26], disulfide connectivity [27,28], half-sphere exposure [29], protein fold recognition and superfamily discrimination [30], ATP binding residues of a protein [31], and catalytic residues [32]. As a result, we also use it to predict bioluminescent proteins.

In this paper, the PSSM of each protein sequence in the constructed dataset is generated against the non-redundant Swiss-Prot database (version 56, released on 22 July 2008) using the PSI-BLAST program with three iterations (−*j* 3) and e-value threshold 0.0001 (−*h* 0.0001). This matrix is composed of *L* × 20 elements, where *L* is the total number of residues in a peptide, the rows of the matrix represent the protein residues and the columns of the matrix represent the 20 amino acids.

**Figure 1:**
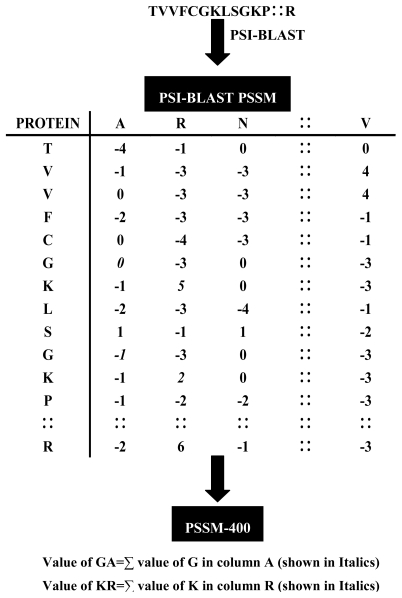
Schematic representation of transformation of each protein sequence into PSSM-400 matrix.

In view of the fact that SVM requires the fixed length feature vectors as their inputs for training [10], we generate a vector of 400 dimensions, called PSSM-400 from the PSSM. PSSM-400 is the composition of occurrences of each type of amino acid corresponding to each type of amino acids in protein sequence. Thus for each column we have a vector of dimension 20. Figure 1 shows the schematic representation of transformation of each protein sequence into PSSM-400. Besides the PSSM-AC encoding strategy, PSSM-400 is also used to encode each protein sequence in this study.

### 2.3. Auto Covariance

Auto covariance (*AC*) is a correlation factor coupling adjacent residues along the protein sequence [11]. It’s a kind of variant of auto cross covariance. As a powerful statistical tool used to analyze sequences of vectors [33], the *AC* transformation has been widely applied in various fields of bioinformatics [34–39]. *AC* variables are able to avoid producing too many variants. In the PSSM-AC encoding strategy, the *AC* transformation is applied to each column of PSSM to incorporate the local sequence-order information. In this study, *AC* is employed to transform the PSSM into equal length vector. Given a protein sequence, *AC* variables describe the average interactions between residues with a series of *lag*. Here, *lag* is the distance between one residue and its neighbors in the protein sequence P. The *AC* variables can be calculated by Equation (1).

(1) $$AC_{lag,j}=(1/(n-lag))\sum_{i=1}^{n-lag}\left(P_{i,j}-(1/n)\sum_{i=1}^{n}P_{i,j}\right)\times (P_{(i+lag),j}-(1/n)\sum_{i=1}^{n}P_{i,j})$$

where *P* represents the PSSM generated by running the PSI-BLAST program, *i* represents the position, *j* represents one descriptor and *n* is the length of the sequence. Thus, the number of *AC* variables *D* can be calculated as *D* = *lg* × *q* (*lg* is the maximum *lag* (*lag* = 1, 2, …, *lg*) and *q* is the number of descriptors). Using Equation 1, each protein sequence can be represented by a vector of *AC* variables, whose length equals to the value of *D*. Here, the value of *q* is 20, which corresponds to the number of the columns of the PSSM. Ultimately, each protein sequence was characterized by the PSSM-AC model.

### 2.4. Support Vector Machine

Support vector machine (SVM) is a popular learning approach mainly used in pattern recognition areas [40–42]. SVM [43] belongs to the family of margin-based classifier and is assumed to be a very powerful method to deal with prediction, classification, and regression problems. SVM looks for the optimal hyperplane which maximizes the distance between the hyperplane and the nearest samples from each of the two classes. Let *x_i_* ∈ *R^n^* be training instance and *y_i_* ∈ {−1, +1} be the corresponding class labels, *i* = 1, ..., *n*. The class label for a new instance *x* can be determined by the sign of the following function.

(2) $$f(x)=\sum_{i=1}^{m}y_{i}\alpha_{i}K(x_{i},x)+b$$

where *m* is the number of training instances, α*_i_* are the obtained by solving a optimization problem on the input instances, and *b* is the bias term. In this paper, LIBSVM package [44] with radial basis kernels (RBF) is used.

(3) $$K(x_{i},x_{j})=\text{exp}(-\gamma {\left\|x_{i}-x_{j}\right\|}^{2})$$

Two parameters, the regularization parameter *C* and the kernel width parameter γ are optimized based on 10-fold cross-validation using a grid search strategy.

### 2.5. Model Construction

**Figure 2:**
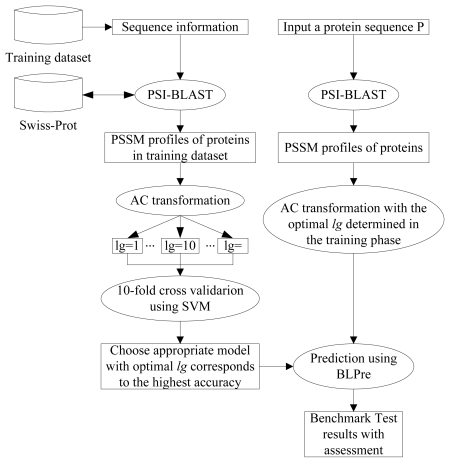
Detailed system flow of the prediction system.

The work flow of the proposed model is described in Figure 2. For the left part of Figure 2, firstly, sequential evolution information in form of PSSM profiles on the training dataset is obtained by PSI-BLAST. Secondly, the *AC* transformation is applied to the obtained PSSM with optional values of *lg* to incorporate local sequence order information. Finally, SVM is applied with ten-fold cross validation. With different *lg*, we can get different prediction models. In this study, we select the one which corresponds to the highest accuracy as the final model. The right part of Figure 2 shows the process of how to predict each one protein sequence using the BLPre predictor.

### 2.6. Performance Evaluation

Ten-fold cross validation [45] is used in this work. The dataset is randomly divided into ten equal sets, out of which nine sets are used for training and the remaining one for testing. This procedure is repeated ten times and the final prediction result is the average accuracy of the ten testing sets. Besides the ten-fold cross validation on the training set, we also utilize independent dataset test [46] to evaluate our model.

Three parameters, sensitivity (*S_n_*), specificity (*S_p_*), and accuracy (*AC*) are used to measure the performance of our model. They are defined by the following formulas:

(4) $$S_{n}=\frac{TP}{TP+FN}$$

(5) $$S_{p}=\frac{TN}{TN+FP}$$

(6) $$AC=\frac{TP+TN}{TP+TN+FP+FN}$$

where *TP*, *TN*, *FP* and *FN* stand for true positive, true negative, false positive and false negative, respectively. Moreover, we create ROC (receiver operating curve) for all of the models in order to evaluate the performance of models using different encoding strategies.

## 3. Results and Discussion

### 3.1. Selecting the Optimal *lg* for the Prediction Model

**Figure 3:**
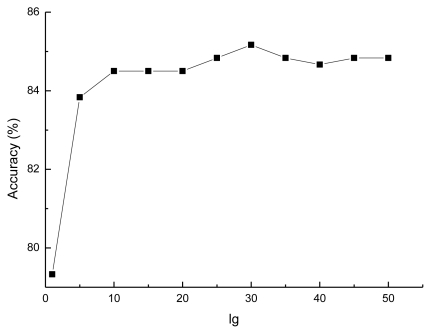
Accuracies of the prediction model with *AC* of different *lg*s.

As mentioned in Section 2.5, for prediction performance, the value of *lg* of *AC* transformation is an important parameter needed to be considered. Generally, the value of *lg* varies in different datasets, and must be smaller than the length of the shortest protein sequence in the corresponding dataset. Since all the protein sequences collected in this paper contain more than 50 amino acids, a series value of *lg*s (*lg* = 1, 5, 10, 15, …, 50) are investigated to construct the optimal prediction model. The results on the training set constructed in this study are presented as Figure 3.

It can be seen in Figure 3, the prediction performance increases from 79.33% to 85.17% when the value of *lg* increases from 1 to 30 and decreases when the value of *lg* is larger than 30. The accuracy of the prediction model becomes stable when the value of *lg* equals 45. It is obvious that the best value of *lg* is 30 corresponding to a peak with accuracy of 85.17%, so that the value of *lg* is set to 30 in the rest of this study.

### 3.2. Comparison with Simple PSI-BLAST Search Method

**Figure 4:**
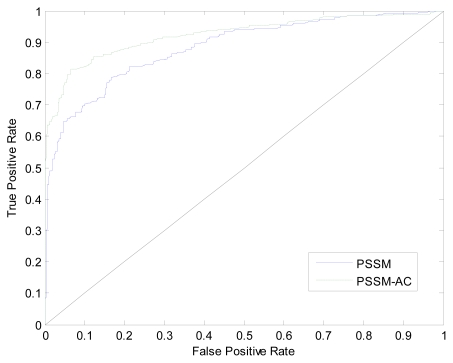
The ROC curves calculated from the ten-fold cross validation of PSSM and PSSM-AC encoding strategies.

In this section, we compare the PSSM-AC encoding strategy with the PSSM-400 encoding strategy mentioned in Section 2.2, thus to highlight the advantage of our prediction model. The results evaluated by ten-fold cross validation on the training dataset are shown in Table 1 and Figure 4. It can be seen in Table 1, compared with the accuracy of 79.32% gained by PSSM-400 method, the accuracy obtained by our method PSSM-AC is 85.17%.

As shown in Figure 4, we achieve the area under the ROC curve (*AUC*) of 0.92, which is significantly better than that of the PSSM-400 method with *AUC* of 0.88. These results indicate that the superior performance of the *AC* transformation encoding when being applied to the PSSM to incorporate the local sequence-order information.

### 3.3. Comparison with Other Methods

In this section, the proposed predictor is further compared with a recently reported predictor BLProt [8] on the training dataset and the independent test dataset. As can be seen in Table 1, our model achieves the accuracy of 85.17%, which is about 5% higher than the BLProt method. The number of bioluminescent proteins and non-bioluminescent proteins in the test dataset are highly imbalanced, and this situation is close to reality. Compared with the accuracy of 80.06% gained by Kandaswamy *et al.* [8], the accuracy obtained by our method is 90.71% which has been significantly improved. The better prediction performance may be credited to the appropriate protein sequence encoding strategy adopted in our prediction model.

## 4. Conclusions

Prediction of bioluminescent proteins could help to discover many still unknown functions and design new commercial and medical applications. Though some researchers have focused on this problem, the accuracy of prediction is still not satisfied. In this study, *AC* is applied to PSSM, and this encoding strategy PSSM-AC could contain both sequential evolution information and the local sequence order information which adequately reflect the local environment during the evolution. The accuracy of our prediction model is higher than those of state-of-the-art bioluminescent proteins prediction tools. Experimental results have shown that our method is very promising and may be a useful supplement tool to existing methods.

## Figures and Tables

**Table 1:** The performance comparison of different encoding strategies on the training dataset.

Method	*S_n_* (%)	*S_p_* (%)	*AC* (%)
PSSM-400	72.00	86.33	79.32
PSSM-AC	79.33	91.00	85.17
BLProt [8]	74.47	84.21	80.00
